# Clinical characteristics, treatment, and outcomes of provoked acute cerebral sinovenous thrombosis in patients <21 years old: findings from the Kids-DOTT Multinational Trial

**DOI:** 10.1016/j.rpth.2024.102605

**Published:** 2024-10-23

**Authors:** Gary M. Woods, Alexandra Miller, Maua Mosha, Christoph Male, Anupam Verma, Nicole Kucine, Christine Sabapathy, Kisha Beg, Sanjay Ahuja, Deepti Raybagkar, Kerry Hege, Clara Lo, Rukhmi Bhat, Thomas Abshire, Neil A. Goldenberg

**Affiliations:** 1 Department of Pediatrics, Emory University and Children’s Healthcare of Atlanta, Atlanta, Georgia, USA; 2 Data Coordinating Center for Pediatric Multicenter Studies, Johns Hopkins All Children’s Hospital Institute for Clinical and Translational Research, St. Petersburg, Florida, USA; 3 Department of Pediatrics, Medical University of Vienna, Vienna, Austria; 4 Department of Pediatrics, University of Utah and Primary Children’s Hospital, Salt Lake City, Utah, USA; 5 Department of Pediatrics, Weill Cornell Medical College and Presbyterian/Weill Cornell, New York, New York, USA; 6 Department of Pediatrics, McGill University Health Centre and The Montreal Children’s Hospital, Montreal, Québec, Canada; 7 Department of Pediatrics, University of Oklahoma College of Medicine and The Children’s Hospital at OU, Oklahoma City, Oklahoma, USA; 8 Department of Pediatrics, Case Western Reserve University School of Medicine and Rainbow Babies & Children’s Hospitals, Cleveland, Ohio, USA; 9 Department of Pediatrics, Drexel University College of Medicine and St. Christopher’s Hospital for Children, Philadelphia, Pennsylvania, USA; 10 Department of Pediatrics, Indiana University School of Medicine and Riley Children’s Health, Indianapolis, Indiana, USA; 11 Department of Pediatrics, Stanford University School of Medicine and Lucile Packard Children’s Hospital, Palo Alto, California, USA; 12 Department of Pediatrics, Northwestern University Feinberg School of Medicine and Ann & Robert H. Lurie Children’s Hospital, Chicago, Illinois, USA; 13 Department of Pediatrics and Medicine, Medical College of Wisconsin and Versiti Blood Research Institute, Milwaukee, Wisconsin, USA; 14 Department of Pediatrics and Medicine, Johns Hopkins University School of Medicine, Baltimore, Maryland, USA

**Keywords:** intracranial, pediatrics, sinus thrombosis, thrombosis, venous thromboembolism

## Abstract

**Background:**

Prospective multicenter data on the treatment and outcomes of children with cerebral sinovenous thrombosis (CSVT) are limited. We aimed to describe the clinical characteristics, treatment strategies, and outcomes of patients with a first-episode of provoked acute CSVT enrolled in the Kids-DOTT trial and compare these features with those of participants with non-CSVT venous thromboembolism (VTE).

**Methods:**

This was a subgroup analysis from the Kids-DOTT trial, a multinational randomized clinical trial on duration of anticoagulation for provoked acute VTE in patients younger than 21 years. Patient and thrombus characteristics, treatments, and outcomes of patients diagnosed with CSVT were compared with those of patients with non-CSVT VTE.

**Results:**

CSVT was diagnosed in 75 of the 532 (14%), 25 of whom received 6 weeks of anticoagulant treatment and 50 received 3 or more months. When compared with non-CSVT VTE, CSVT was more likely to occur in neonates and young children, associated with infection in general and acute head/neck infection in particular, and less likely to be related to central venous catheter. No patient in either group developed symptomatic recurrent VTE or clinically relevant bleeding, and there was no significant difference in rates of complete thrombus resolution between the 2 treatment durations.

**Conclusion:**

CSVT is most common in neonates and young children and those with acute head and neck infections. A 6-week anticoagulation treatment course appears to be safe (no clinically relevant bleeding) and effective (no difference in symptomatic recurrent VTE) for provoked acute pediatric CSVT. Nevertheless, given the nature of a subpopulation analysis, these findings should be interpreted with caution.

## Essentials

• Data on treatment and outcomes of cerebral sinovenous thrombosis (CSVT) in children are limited.
• This is a subgroup (CSVT) analysis from the Kids-DOTT trial.
• CSVT is most common in neonates and young children and with head and neck infections.
• Six weeks of anticoagulation appears to be safe and effective for acute provoked pediatric CSVT.

## 1 Introduction

Cerebral sinovenous thrombosis (CSVT), or venous thromboembolism (VTE) of the cerebral or dural veins and sinuses, is a rare condition seen in about 0.7 per 100,000 children annually [1]. CSVT accounts for about 9% of all pediatric VTE with up to 50% reported in the neonatal age group [2,3]. The vast majority of pediatric CSVTs have an identifiable provoking risk factor, but these are age dependent and variable [3,4]. Common risk factors for acute provoked neonatal CSVT include perinatal distress and infections, whereas acute infections, dehydration, and severe iron deficiency are commonly associated with pediatric CSVT [3].

Few multicenter clinical trials have reported specifically on pediatric CSVT treatments and outcomes, and data from prospective multicenter observational studies are also limited. The standard of care for the treatment of pediatric CSVT, even in the presence of hemorrhage secondary to venous congestion, is therapeutic anticoagulation, a recommendation extrapolated from evidence in adults [[5], [6], [7]]. An analysis of the Pediatric Stroke Study registry demonstrated that nearly 80% of 410 enrolled patients with CSVT received anticoagulation and that administration (vs nonadministration) of anticoagulation was associated with favorable outcomes [4]. Consensus guidelines currently suggest 3 months of anticoagulation for acute provoked pediatric CSVT [[8], [9], [10], [11], [12], [13]].

Recently, the primary findings of the Kids-DOTT trial, an NHLBI-sponsored multinational randomized clinical trial (RCT; NCT00687882) on duration of anticoagulation for provoked acute VTE in patients younger than 21 years, were published and revealed noninferiority of a 6-week course of anticoagulation, when compared with those of a 3-month course, for provoked VTE in patients younger than 21 years [14]. The objective of this work was to describe the clinical characteristics and treatment strategies of the patients with provoked acute CSVT enrolled in the Kids-DOTT trial and to compare these features with those of Kids-DOTT participants with non-CSVT VTE. We also aimed to describe and compare the outcomes of the patients with provoked acute CSVT who received 6 weeks compared with those who received at least 3 months of anticoagulation.

## 2 Methods

### 2.1 Study design and participant eligibility

Utilizing data from the Kids-DOTT trial, a prespecified subgroup analysis was performed on the patients specifically with a first-episode acute provoked CSVT. There were 42 participating centers across North America, Europe, and Australia that obtained institutional review board approval and written informed consent and assent where appropriate to enroll patients younger than 21 years with an acute provoked VTE [14]. Patients were enrolled no more than 30 days from VTE diagnosis. Those with natural anticoagulant deficiencies, lupus, active cancer, proximal pulmonary embolus, previous VTE, or thrombolytic intervention for the index VTE were excluded. Anticoagulation was initiated, and patients were treated for 6 weeks, at which point repeat imaging of index VTE was obtained. Patients who met criteria for randomization (lack of complete veno-occlusion or persistently positive antiphospholipid antibodies [aPL]) were then randomly assigned at a ratio of 1:1 to receive 6 weeks or 3 months total duration of anticoagulation. Those ineligible for randomization (persistently positive aPL or complete veno-occlusion at 6 weeks) were followed up in nonrandomized cohort arms running in parallel to the randomized arms and received a total duration of anticoagulation of at least 3 months.

### 2.2 Data collection

Baseline patient and thrombus characteristics, treatments, and outcomes were recorded prospectively. Symptomatic recurrent VTE between 10 days and 1 year from the diagnosis of the index VTE was the primary efficacy outcome definition, but for trial design purposes, any patient developing a recurrent VTE prior to the 6-week follow-up visit were withdrawn from the trial per protocol. Clinically relevant bleeding (CRB), comprising major bleeding and clinically relevant nonmajor bleeding, was the primary safety outcome. Clinical efficacy and safety outcomes data were also collected prospectively. Efficacy and safety outcomes were defined using internationally standardized outcome definitions for pediatric thrombosis research [15].

### 2.3 Statistical analyses

Variables were compared between patients in whom the index VTE was CSVT (CSVT group) and all others (non-CSVT VTE group). Continuous variables were compared between groups utilizing the Wilcoxon rank sum test. Categorical variables were compared between groups using chi-squared or Fisher exact test as appropriate. For all statistical analyses, a *P* value of <.05 was considered statistically significant. All statistical analyses were performed using R software, version 4.3.0 (R Foundation for Statistical Computing), and SAS, version 9.4.

## 3 Results and Discussion

Among 532 patients enrolled in the Kids-DOTT trial, the index VTE was a CSVT in 75 (14%) patients. Baseline patient and thrombus characteristics for the CSVT and non-CSVT VTE groups are shown in Table 1. Patients with CSVT were more likely to be preadolescent than those with non-CSVT VTE (81% vs 63%; *P* = .002).

**Table 1:** Baseline characteristics of the Kids-DOTT study population.

Variable	Overall, *N* = 532^a^	CSVT, *n* = 75^a^	Non-CSVT VTE, *n* = 457^a^	*P*^b^
Age (y)	8.0 (1.0, 15.0)	6.6 (2.3, 11.3)	8.5 (0.9, 15.6)	.10
Age group				**.002**
Neonate (0–29 d)	23 (4)	6 (8)	17 (4)	
Child (30 d to <13 y)	326 (61)	55 (73)	271 (59)	
Teen (≥13 y)	183 (34)	14 (19)	169 (37)	
Sex (% male)	283 (53)	47 (63)	236 (52)	.076
Overweight/obese	123/342 (36)	20/57 (35)	103/285 (36)	.90
Provoking factors				
Central venous catheter	235/472 (50)	4/64 (6)	231/408 (57)	**<.001**
Hospitalization within previous 30 d	6/472 (1)	0/64 (0)	6/408 (2)	>.90
Trauma/surgery within previous 30 d	91/472 (19)	9/64 (14)	82/408 (20)	.20
Autoimmune/inflammatory condition	14/472 (3)	1/64 (2)	13/408 (3)	.70
Estrogen/other prothrombotic medication	29/472 (6)	5/64 (8)	24/408 (6)	.60
Congenital/acquired cardiac disease	3/472 (1)	0/64 (0)	3/408 (1)	>.90
Infection	143/472 (30)	37/65 (57)	106/407 (26)	**<.001**
Head/neck	31/472 (7)	23/65 (35)	8/407 (2)	**<.001**
Non–head/neck	35/472 (7)	0/65 (0)	35/407 (9)	**.009**
Unknown site	77/472 (16)	14/65 (22)	63/407 (16)	.20

The bolded are statistically significant findings.

CSVT, cerebral sinovenous thrombosis; VTE, venous thromboembolism.

a Median (IQR); *n* (%).

b Wilcoxon rank sum test; Fisher exact test; Pearson chi-squared test.

When comparing VTE provoking factors, the frequency of infection (57% vs 26%; *P* < .001) and specifically infection of the head/neck (35% vs 2%; *P* < .001) was significantly higher in the CSVT group compared with that in the non-CSVT VTE group. The presence of an acute infection has been previously described as a common risk factor for the development of acute VTE, including CSVT [3,13]. Our data confirm these observations by revealing statistically significant increased odds of infection, head/neck infection in particular, among pediatric patients with CSVT when compared with those with non-CSVT VTE. Unsurprisingly, non-CSVT VTE were significantly more likely to be associated with a central venous catheter when compared with CSVT, although 4 patients with CSVT and internal jugular (IJ) vein involvement did have a central venous catheter present in the subclavian/IJ region. Intracranial hemorrhage (ICH) at diagnosis of CSVT (based on baseline imaging) was not prespecified as a variable of interest, but was assessed post hoc in 59 patients. Among these, ICH was present at CSVT diagnosis in 3 patients (5%). Among 75 patients with CSVT in the Kids-DOTT trial, 25 (33%) were randomized to receive 6 weeks, 34 (45%) were randomized to receive 3 months, and 16 (21%) were nonrandomly assigned to receive 3 months of anticoagulation due to continued complete veno-occlusion (*n* = 12 [16%]) or persistence of aPL (*n* = 4 [5%]) at 6 weeks (Table 2). Some patients did require medical and/or procedural interventions for increased intracranial pressure, including acetazolamide use in 6 of the 41 (14%), cerebrospinal fluid shunt/drainage in 4 of the 40 (10%), and anticonvulsant therapy in 12 of the 38 (32%) patients, although data were incomplete. Patients who required medical and/or surgical intervention for increased intracranial pressure were significantly more likely to have persistent thrombosis at 6 weeks (*P* = .034).

**Table 2:** CSVT anatomic sites, treatments, and outcomes in the Kids-DOTT study CSVT subpopulation (*n* = 75).

Variable	Frequency (%)
CSVT anatomic site	
Sigmoid sinus: left-sided	7/44 (16)
Sigmoid sinus: right-sided	6/44 (14)
Superior sagittal sinus	10/44 (23)
Transverse sinus: left-sided	10/44 (23)
Transverse sinus: right-sided	10/44 (23)
Involvement of internal jugular vein	30/75 (40)
Intracranial hemorrhage at diagnosis	3/59 (5)
Duration of anticoagulation	
6 wk	25/75 (33)
≥3 mo	50/75 (67)
Symptomatic recurrent VTE	0/75 (0)
Clinically relevant bleeding	0/75 (0)
Acetazolamide use	6/41 (15)
CSF shunt/drainage intervention	4/40 (10)
Anticonvulsant administration	12/38 (32)

CSF, cerebrospinal fluid; CSVT, cerebral sinovenous thrombosis; VTE, venous thromboembolism.

None of the patients developed symptomatic recurrent VTE or CRB within 1 year of the index CSVT. At the time of randomization at 6 weeks of therapy, there was no statistically significant difference in complete thrombus resolution rate when comparing the 6-week and the combined randomized and nonrandomized 3-month duration of therapy arms (10/21; 48%, vs 20/47; 43%; *P* = 0.69). When evaluating for potential associations between CSVT anatomic location and thrombosis resolution versus persistence at 6 weeks, CSVT with left-sided transversal (*P* = .033), sigmoidal (*P* = .036), or IJ (*P* = .017) vein involvement was significantly more likely to persist at the 6-week evaluation (Table 3). It is important to acknowledge that incomplete data, as well as the potential for bias and confounding, could have affected our statistical analysis.

**Table 3:** CSVT anatomic site and association with thrombus persistence at 6 weeks of therapy.

CSVT anatomic site	*P*^a^
Superior sagittal sinus	.72
Inferior sagittal sinus	1
Straight sinus	1
Left transverse sinus	**.03**
Right transverse sinus	1
Left sigmoid sinus	**.04**
Right sigmoid sinus	.53
Left internal jugular	**.02**
Right internal jugular	.69

The bolded are statistically significant findings.

a Fisher's exact test.

This analysis from the multinational Kids-DOTT RCT and parallel observational cohorts reveals that children with CSVT are significantly more likely to be preadolescent and to have acute head/neck infection as a provoking factor than those with non-CSVT VTE. Among 75 patients with CSVT, there were no symptomatic recurrent VTE or CRB events within 1 year and no difference in complete CSVT resolution rates over the monitored time. Previous recommendations for acute CSVT anticoagulation suggested that a 3- to 6-month course was reasonable, which was largely extrapolated from adult data [3]. Based on this subanalysis, we have no evidence to suggest that the Kids-DOTT findings on duration of therapy for provoked pediatric VTE do not apply to the subpopulation with provoked CSVT. It is worth noting that, after completion of a 6-week primary treatment course of anticoagulation for provoked VTE in patients younger than 21 years, we believe that secondary prophylaxis (eg, low dose) may be warranted in some cases, such as those patients who are still being treated to control an underlying comorbidity (eg, continued antibiotics to achieve resolution of infection that has persisted beyond 6 weeks). The role of secondary anticoagulation following completion of a primary treatment course in children with provoked VTE is being evaluated in a separate secondary analysis from the Kids-DOTT Investigator Group.

Patients with CSVT may present with an associated ICH leading to hesitation in initiating anticoagulation. Additionally, infections of the head and neck area have been associated with an increased risk of intracerebral hemorrhage [16]. Findings from the International Pediatric Stroke Study have shown that anticoagulation use is associated with favorable outcomes in children with CSVT, and recent consensus guidelines suggest using anticoagulation in pediatric patients with CSVT despite the presence of hemorrhage [4,8]. Our data show a low risk for CRB in a similar pediatric population with CSVT treated with anticoagulant therapy. This suggests that anticoagulation can be safely used for pediatric CSVT treatment, even when associated with an underlying head/neck infection. Moreover, in pediatric patients with acute provoked CSVT without persistent risk factors, a 6-week course of anticoagulation appears to be safe with regards to recurrent VTE and CRB.

Strengths of our study include a prospective design, multinational site participation, detailed data capture on anticoagulant agent administration, and a blinded, independent, central adjudication of primary efficacy and safety outcomes that employ internationally standardized outcome definitions. Potential limitations of the present work include the relatively small sample size of the CSVT subpopulation and the lack of data on neurologic and neuropsychological outcomes. Given the nature of a subpopulation analysis, our findings should be interpreted with caution. Additionally, in regard to risks of bleeding and the low rate of CRB observed in this study, selection bias cannot be absolutely ruled out. However, for a trial that evaluated a shortened duration of therapy, one would expect that if there were a selection bias, it would have resulted in patients with higher-than-average (rather than lower) a priori risk for bleeding to be enrolled. Furthermore, our overall rates of CRB and recurrent VTE among patients with CSVT in the Kids-DOTT RCT are concordant with those of the pediatric direct oral anticoagulant randomized clinical trials [17,18].

Notwithstanding these potential limitations, this analysis from the Kids-DOTT multinational RCT and parallel-cohort study demonstrate that acute provoked CSVT is more frequent in preadolescents with acute head/neck infection as an important provoking factor compared with those with non-CSVT VTE. A 6-week course of anticoagulation is associated with low risk of recurrent VTE and CRB and our data suggest previously published findings in the overall VTE population of the Kids-DOTT RCT can be applied to the subpopulation with provoked CSVT.
